# Supplementary material for: Pan-serotype dengue virus inhibitor JNJ-A07 targets NS4A-2K-NS4B interaction with NS2B/NS3 and blocks replication organelle formation
Source: Nat Commun. 2024 Jul 19;15:6080. doi: 10.1038/s41467-024-50437-3 (PMC11271582; doi:10.1038/s41467-024-50437-3)
Supplement: Supplementary file 1 — Supplementary Information [file 41467_2024_50437_MOESM1_ESM.pdf]

Supplementary information

Pan-serotype dengue virus inhibitor JNJ-A07 targets NS4A-2K-NS4B interaction with NS2B/NS3 and blocks replication organelle formation

Dominik Kiemel, Ann-Sophie Helene Kroell, Solène Denolly, Uta Haselmann, Jean-François Bonfanti, Jose Ignacio Andres, Brahma Ghosh, Peggy Geluykens, Suzanne J. F. Kaptein, Lucas Wilken, Pietro Scaturro, Johan Neyts, Marnix Van Loock, Olivia Goethals, Ralf Bartenschlager*

*Corresponding author: ralf.bartenschlager@med.uni-heidelberg.de

This PDF contains supplementary methods, figures and references.

Supplementary Methods

Synthesis of Compound A and Compound B

General chemistry experimental details

All commercial reagents were used without further purification. Dry solvents were used.

NMR experiments were carried out using a Bruker Avance III 400 or 500 spectrometer, at ambient temperature (298.6 K), using internal deuterium lock, and equipped with reverse double-resonance (^{1}H, ^{13}C) probe head with z gradients, and operating at 400 MHz or 500 MHz for the proton, and 100 Hz or 90 Hz for the carbon, respectively. Chemical shifts (d) are reported in parts per million (ppm). *J* values are expressed in Hz. The following abbreviations were used for multiplicities: s = singlet, d = doublet, t = triplet, q = quartet, m = multiplet, dd = doublet of doublets, td = triple doublet, dt = double triplet, and br = broad.

LC-MS analyses were performed using a liquid chromatography pump, a diode-array (DAD) or a UV detector and a column as specified in the respective methods. If necessary, additional detectors were included (see methods below). The flow from the column was brought to the Mass Spectrometer (MS) which was configured with an atmospheric pressure ion source. Data acquisition was performed with appropriate software. If not specified otherwise, the reported molecular ion corresponds to the $[M+H]^+$ (protonated molecule). For molecules containing atoms with multiple isotopic patterns (Br, Cl), the reported value is the one obtained for the lowest isotope mass. All results were obtained with experimental uncertainties that are commonly associated with the method used. “SQD” means Single Quadrupole Detector, “DAD” Diode Array Detector.

LCMS1 conditions: reversed-phase UPLC-DAD and SQD was carried out on a BEH C18 column (1.7 µm, 2.1 × 50 mm) from Waters with a flow rate of 1 mL/min at 50 °C. The gradient conditions used were as follows: 95 % A (6.5 mM CH_3COONH_4 in 95 % H_2O + 5 % CH_3CN), 0 % B (CH_3CN), to 40 % A in 1.2 min, to 5 % A in 0.6 min, held for 0.2 min, with a total run time of 2 min.

LCMS2 conditions: reversed-phase UPLC-DAD and SQD was carried out on a BEH C18 column (1.7 µm, 2.1 × 100 mm) from Waters with a flow rate of 0.34 mL/min at 40 °C. The gradient conditions used were as follows: 84.2 % A (7 mM CH_3COONH_4 in 95 % H_2O + 5 % CH_3CN), 15.8 % B (CH_3CN), to 10.5 % A in 2.18 min, held for 1.94 min, back to 84.2 % A, held for 0.73 min, with a total run time of 6.2 min.

Chiral analytical SFC measurements were performed using an analytical Supercritical Fluid Chromatography (SFC) system composed by a binary pump for delivering carbon dioxide (CO_2) and modifier, an autosampler, a column oven, a diode array detector equipped with a high-pressure flow cell standing up to 400 bars. If configured with a Mass Spectrometer (MS)

the flow from the column was brought to the MS. Data acquisition was performed with appropriate software.

SFC conditions: SFC was carried out on a Chiralpak AD Daicel column (10 µm, 4.6 x 250 mm) with a flow rate of 3.0 mL/min. The mobile phase was 70 % CO_2 with 30 % EtOH in isocratic mode.

Optical rotations were measured on a Perkin-Elmer 341 polarimeter with a sodium lamp. The specific optical rotation $[\alpha]_D^{20}$ is reported in degrees.

The following description of the synthesis pathway of Compound A and Compound B is shown schematically in Supplementary Figure 2.

2-(4-Chloro-2-methoxyphenyl)-2-((3-methoxy-5-(2-methoxyethoxy)phenyl)amino)-1-(5-(trifluoromethoxy)-1H-indol-3-yl)ethan-1-one

DIPEA (149 µL, 0.865 mmol, 2 eq.) was added to a mixture of 2-bromo-2-(4-chloro-2-methoxyphenyl)-1-(5-(trifluoromethoxy)-1*H*-indol-3-yl)ethan-1-one* (200 mg, 0.432 mmol), and 3-methoxy-5-(2-methoxyethoxy)aniline (CAS [725237-15-0], 256 mg, 1.300 mmol, 3 eq.), in s-BuOH at room temperature. The reaction mixture was stirred at 55 °C for 2 h. After cooling, the reaction mixture was treated with a 1 M aqueous HCl and extracted with DCM. The organic layer was washed with brine, dried on Na_2SO_4, filtered, and concentrated *in vacuo*. The residue was purified by flash column chromatography (silica, [7 M NH_3 in MeOH in DCM 1:9]/DCM from 0/100 to 98/2) to yield 2-(4-chloro-2-methoxyphenyl)-2-((3-methoxy-5-(2-methoxyethoxy)phenyl)amino)-1-(5-(trifluoromethoxy)-1*H*-indol-3-yl)ethan-1-one (194 mg, 87 % pure, quantitative), which was used without further purification in the next step.

*Intermediate **3** in Supplementary Reference [1]

^{1}H NMR (500 MHz, $CDCl_3$) δ (ppm): 3.43 (s, 3 H), 3.67 (s, 3 H), 3.69 - 3.73 (m, 2 H), 3.94 (s, 3 H), 3.99 - 4.05 (m, 2 H), 5.49 (br s, 1 H), 5.82 - 5.86 (m, 1 H), 5.87 (br s, 2 H), 6.05 (s, 1 H), 6.82 - 6.87 (m, 2 H), 7.12 (dd, *J*=8.8, 1.6 Hz, 1 H), 7.27 (d, *J*=8.4 Hz, 1 H), 7.31 (d, *J*=8.7 Hz, 1 H), 8.10 (d, *J*=3.2 Hz, 1 H), 8.27 (s, 1 H), 8.98 (br s, 1 H); LC/MS: R_t 1.59 min, $[M+H]^+$ 579.1; purity 87 % (method LCMS1)

(+)-1-(1-(2-(3-(But-3-yn-1-yl)-3H-diazirin-3-yl)ethyl)-5-(trifluoromethoxy)-1H-indol-3-yl)-2-(4-chloro-2-methoxyphenyl)-2-((3-methoxy-5-(2-methoxyethoxy)phenyl)amino)ethan-1-one (Compound A) and (-)-1-(1-(2-(3-(but-3-yn-1-yl)-3H-diazirin-3-yl)ethyl)-5-(trifluoromethoxy)-1H-indol-3-yl)-2-(4-chloro-2-methoxyphenyl)-2-((3-methoxy-5-(2-methoxyethoxy)phenyl)amino)ethan-1-one (Compound B)

Sodium hydride (60 % in mineral oil, 31 mg, 0.806 mmol, 1 eq.) was added to a solution of 2-(4-chloro-2-methoxyphenyl)-2-((3-methoxy-5-(2-methoxyethoxy)phenyl)amino)-1-(5-(trifluoromethoxy)-1*H*-indol-3-yl)ethan-1-one (598 mg, 0.806 mmol) in dry DMF at 0 °C under

nitrogen atmosphere. The reaction mixture was stirred at 0 ºC for 15 min. Then 3-(but-3-yn-1-yl)-3-(2-iodoethyl)-3*H*-diazirine (CAS [1450754-38-7], 200 mg, 0.806 mmol, 1 eq.) was added and the resulting mixture was stirred at room temperature for 2 h. The reaction mixture was cooled down to 0 ºC, diluted with EtOAc, and quenched with water. The organic layer was separated and the aqueous layer was extracted with EtOAc (3x). The combined organic layers were dried (Na_2SO_4), filtered, and concentrated *in vacuo*. The crude product was purified by flash column chromatography (silica; [7 M NH_3 in MeOH in DCM 1:9]/DCM from 0/100 to 2/98). The desired fractions were collected and concentrated *in vacuo*. The residue was triturated in diethyl ether to give a white solid. This racemate was separated into its stereoisomers by chiral SFC (Stationary phase: Chiralpak AD-H 5 µm 250 x 30 mm, Mobile phase: 70 % CO_2, 30 % EtOH) to afford (-)-1-(1-(2-(3-(but-3-yn-1-yl)-3*H*-diazirin-3-yl)ethyl)-5-(trifluoromethoxy)-1*H*-indol-3-yl)-2-(4-chloro-2-methoxyphenyl)-2-((3-methoxy-5-(2-methoxyethoxy)phenyl)amino)ethan-1-one (**Compound B**, 102 mg, yield: 18 %) as the first eluting enantiomer and (+)-1-(1-(2-(3-(but-3-yn-1-yl)-3*H*-diazirin-3-yl)ethyl)-5-(trifluoromethoxy)-1*H*-indol-3-yl)-2-(4-chloro-2-methoxyphenyl)-2-((3-methoxy-5-(2-methoxyethoxy)phenyl)amino)ethan-1-one (**Compound A**, 120 mg, yield: 21 %) as the second eluting enantiomer, obtained both as white solids.

Compound A:

^{1}H NMR (500 MHz, DMSO-d_6) d (ppm) 1.50 (t, 2H, *J*=7.4 Hz), 1.9-2.0 (m, 5H), 2.80 (t, 1H, *J*=2.5 Hz), 3.28 (s, 3H), 3.58 (t, 2H, *J*=4.6 Hz), 3.61 (s, 4H), 3.9-4.0 (m, 2H), 3.98 (s, 3H), 4.28 (br t, 2H, *J*=7.3 Hz), 5.74 (s, 1H), 5.93 (br d, 2H, *J*=8.2 Hz), 6.15 (d, 1H, *J*=8.2 Hz), 6.42 (d, 1H, *J*=7.9 Hz), 6.97 (dd, 1H, *J*=1.7, 8.4 Hz), 7.09 (d, 1H, *J*=1.6 Hz), 7.29 (br d, 1H, *J*=8.8 Hz), 7.37 (d, 1H, *J*=8.2 Hz), 7.75 (d, 1H, *J*=8.8 Hz), 8.08 (s, 1H), 8.68 (s, 1H); ^{13}C NMR (101 MHz, DMSO-d_6) d (ppm) 12.98, 27.20, 31.43, 33.12, 42.01, 55.16, 56.63, 58.60, 66.87, 70.85, 72.30, 83.50, 90.10, 92.45, 92.70, 112.04, 113.13, 113.70, 113.93, 117.36, 119.52, 121.13, 126.76, 127.12, 129.61, 133.60, 135.34, 138.98, 144.76, 149.09, 157.51, 160.62, 161.46, 191.25; LC/MS: R_t 3.77 min, $[M+H]^+$ 699.4; purity 99 % (method LCMS2); Chiral SFC: R_t 1.73 min, $[M+H]^+$ 699, chiral purity 100 %; $[\alpha]_D^{20}$: +42.6° (589 nm, c 0.265 w/v %, DMF, 20 °C)

Compound B:

^{1}H NMR (DMSO-d_6, 500 MHz) d (ppm) 1.50 (t, 2H, *J*=7.4 Hz), 1.9-2.0 (m, 4H), 2.79 (t, 1H, *J*=2.5 Hz), 3.3-3.3 (m, 3H), 3.6-3.6 (m, 2H), 3.62 (s, 3H), 3.9-4.0 (m, 2H), 3.98 (s, 3H), 4.28 (s, 2H), 5.74 (t, 1H, *J*=1.9 Hz), 5.93 (br d, 2H, *J*=7.9 Hz), 6.15 (d, 1H, *J*=8.2 Hz), 6.41 (d, 1H, *J*=8.2 Hz), 6.97 (dd, 1H, *J*=1.9, 8.2 Hz), 7.09 (d, 1H, *J*=1.9 Hz), 7.28 (dd, 1H, *J*=1.9, 8.8 Hz), 7.37 (d, 1H, *J*=8.2 Hz), 7.75 (d, 1H, *J*=8.8 Hz), 8.08 (s, 1H), 8.67 (s, 1H); ^{13}C NMR (101 MHz, DMSO-d_6) d (ppm) 11.90, 26.13, 30.36, 32.05, 40.94, 54.09, 55.55, 57.53, 65.80, 69.78, 71.23, 82.43, 89.02, 91.38, 91.62, 110.97, 112.05, 112.86, 116.29, 118.45, 120.06, 120.99, 125.68, 126.04, 128.54, 132.52, 134.27, 137.91, 143.70, 143.70, 148.01, 156.44, 159.55, 160.40, 190.18; LC/MS: R_t 3.77 min, $[M+H]^+$ 699.4; purity 100 % (method LCMS2); Chiral SFC: R_t 1.01 min, $[M+H]^+$ 699, chiral purity 100 %; $[\alpha]_D^{20}$: -44.6° (589 nm, c 0.316 w/v %, DMF, 20 °C)

Supplementary Figures

a

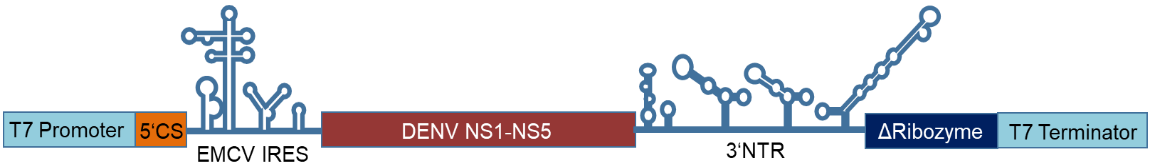

b

Compound	Structure	Antiviral EC_{50} (µM)	Toxicity CC_{50} (µM)	SI
JNJ-A07	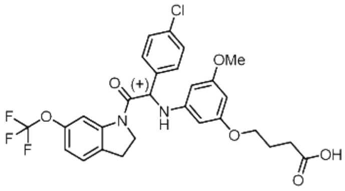	0.0001 ± 0.00007	13 ± 1.1	130,000
Compound A	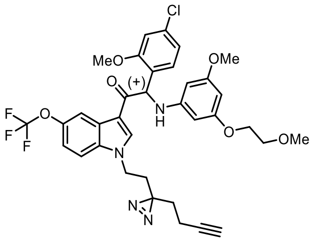	0.015 ±0.006	>25	>1,600
Compound B	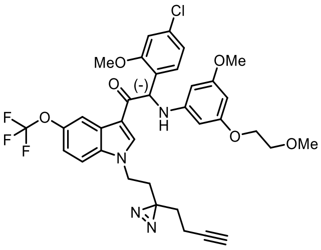	0.9 ±0.6	>25	>28
NITD-688	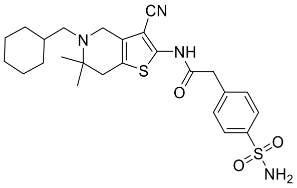	0.09 ± 0.07	>41 ± 13	>456

Supplementary Fig. 1 | Overview of the pIRO-system and compounds used in this study. a, Schematic depiction of the pIRO-D system. Transfection of cells expressing the DNA-dependent RNA polymerase of bacteriophage T7 with pIRO-D plasmids allows the expression of the non-structural DENV proteins (NS1-NS5). Upstream of the coding region is the T7 RNA polymerase promoter, a short fragment of the capsid region containing the 5′ cyclization sequence (CS) as well as an internal ribosome entry site (IRES) of the encephalomyocarditis

virus (EMCV) to enable high-level RNA translation. Downstream of the coding region are the 3'NTR, which plays an important role in DENV VPs formation, the ribozyme of the hepatitis D virus (Δ) to generate the authentic 3' end and the T7 terminator. Expression of the pIRO-D construct induces VPs; the pIRO-Z system works analogously for ZIKV. **b,** Table showing the structures and key parameters of compounds used in this study. For JNJ-A07 and NITD-688, data represent average values ± standard deviations from at least five independent experiments using DENV-2/16681 on Vero cells. For Compound A and Compound B, EC_{50} and CC_{50} were derived from three independent experiments. Selectivity index (SI) was calculated by dividing the average CC_{50} value by the average EC_{50} value. EC_{50}, 50% effective concentration; CC_{50}, 50% cytotoxic concentration.

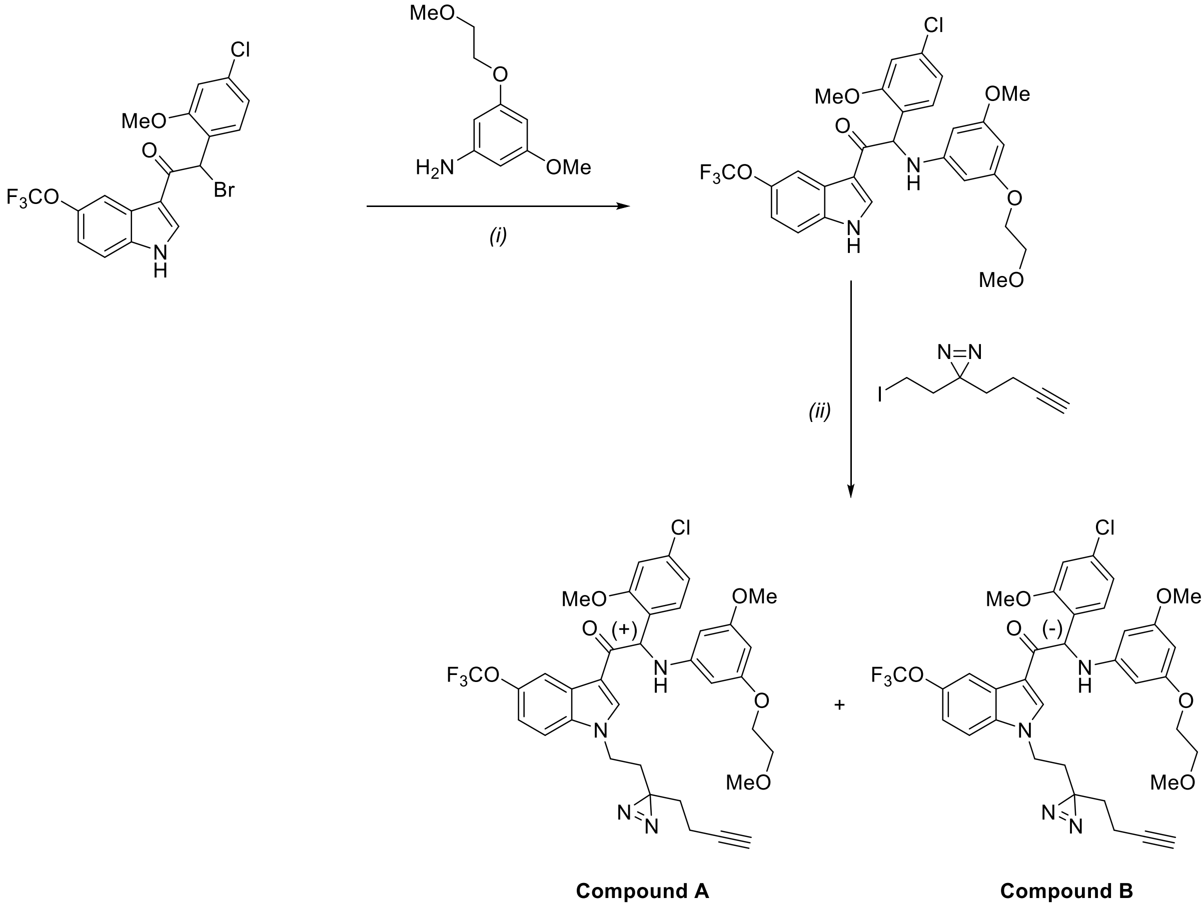

Supplementary Fig. 2 | Synthesis of Compound A and Compound B.
(i) DIPEA, s-BuOH, 55 °C, 2 h; (ii) NaH, DMF, 0 °C to r.t., 2 h, followed by chiral separation.

a

NS4B mutation	Replication fitness*		Resistance to JNJ-A07*		Resistance to Compound A†	
	Amplification ratio (72h vs. 4h)	Fold change	EC_{50} (μM)	Fold change	EC_{50} (μM)	Fold change
None (WT)	12.0 ± 2.9	/	0.003 ± 0.001	/	0.058 ± 0.007	/
S85L	2.0 ± 0.9	0.2	0.03 ± 0.003	6	0.477 ± 0.028	8
V91A	11.2 ± 4.4	0.9	0.1 ± 0.07	63	>25	>464
L94F	25.8 ± 6.9	2.2	1 ± 0.1	950	>4.433	>80
T108I	8.3 ± 2.5	0.7	0.02 ± 0.02	10	0.88 ± 0.058	16
A137T	12.2 ± 10.3	1	0.005 ± 0.0001	1	0.088 ± 0.007	2
T216N	3.5 ± 1.8	0.3	0.1 ± 0.009	24	9.250	>168

b

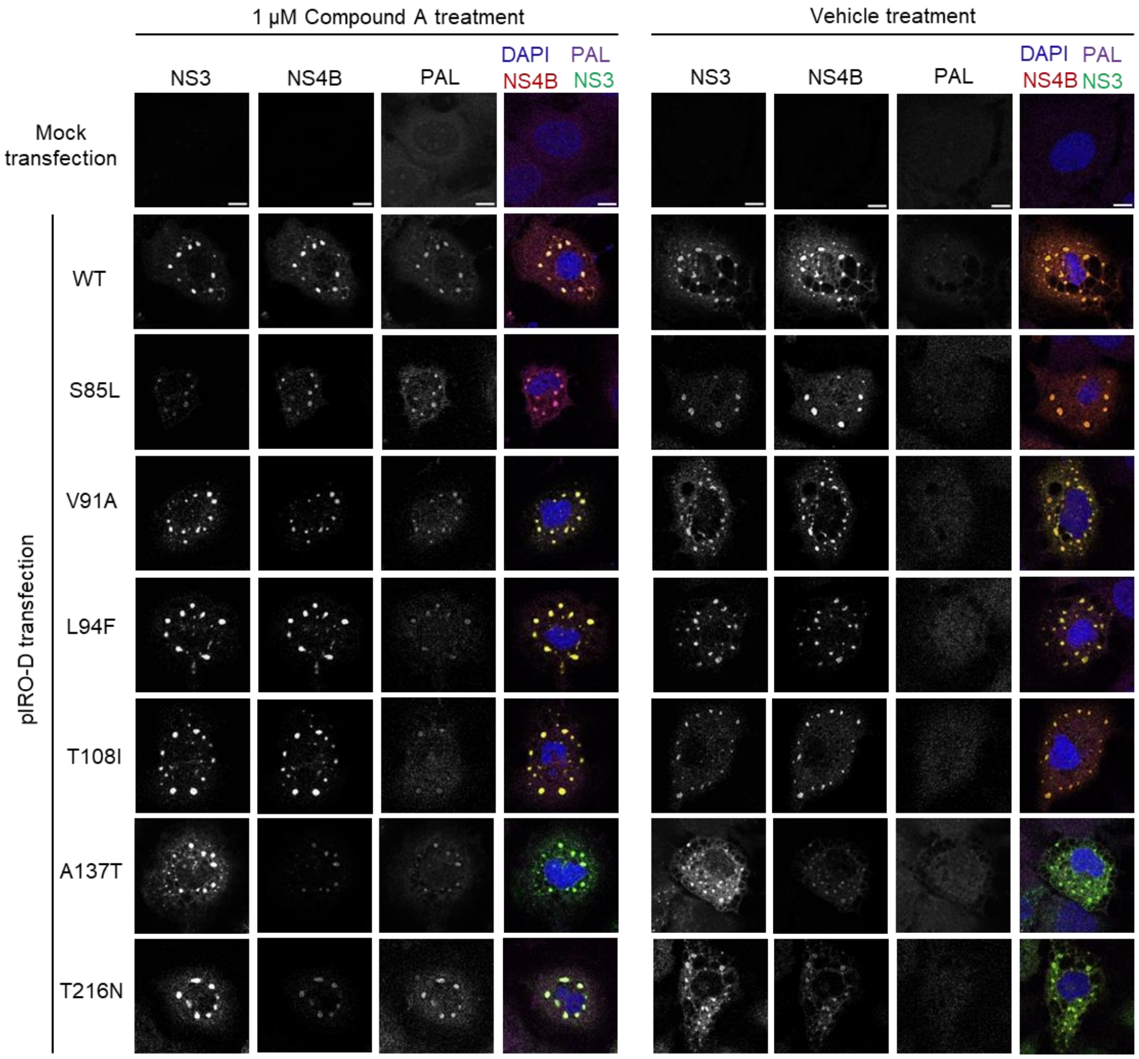

Supplementary Fig. 3 | Properties of NS4B inhibitor resistance mutants and photoaffinity labeling of cells expressing compound-resistant polyproteins.

a, Compilation of important parameters of NS4B resistance mutations. The effect of resistance mutations in NS4B on replication fitness was determined by using subgenomic DENV *Renilla* luciferase reporter replicons (sgDVs-R2A; strain DENV 2/16681). Replication efficiency was

determined by normalizing the luciferase signal of the 72 h time point, which marks the peak in signal intensity in all samples, to the 4 h time point that reflects transfection efficiency*. For each mutant, fold change of replication fitness is given relative to WT. The level of compound resistance mutations to JNJ-A07* and Compound A† was determined using sgDVs-R2A replicons. Fold change in EC_{50} of compound resistance was calculated as the average ratio between the EC_{50} value for a mutant strain relative to the EC_{50} value for the WT tested in the same experiment. Data represent mean values ± standard deviation of a minimum of 3 measurements from at least 2 independent experiments. *Data recapitulated from Supplementary Reference [2]; †Data generated in this study. **b,** IF-panel of Huh7/Lunet-T7 cells transfected with pIRO-D WT plasmid or mutants thereof containing indicated resistance mutations in NS4B. Experimental procedure as depicted in Fig. 1a. Images were obtained by confocal microscopy at 60x magnification. Scale bar = 10 µm.

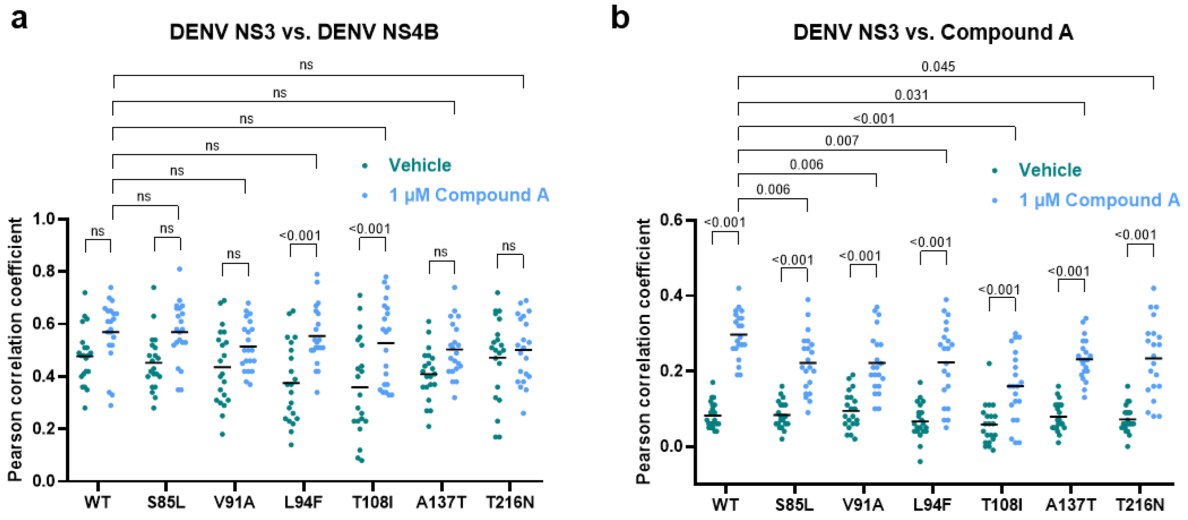

Supplementary Fig. 4 | NS4B mutations reduce colocalization between NS3 and Compound A, but not between NS3 and NS4B.

a-b, The data set for which colocalization between NS4B and Compound A is shown (Fig. 1f), was analyzed analogously to determine the colocalization between NS4B and NS3 (a), and between NS3 and Compound A (b). Pearson correlation coefficients based on 7 cell profiles for each of the 3 independent experiments are plotted with mean (black horizontal line). Statistical significance was assessed using a two-way ANOVA test and Šidák's multiple comparisons. ns = non-significant.

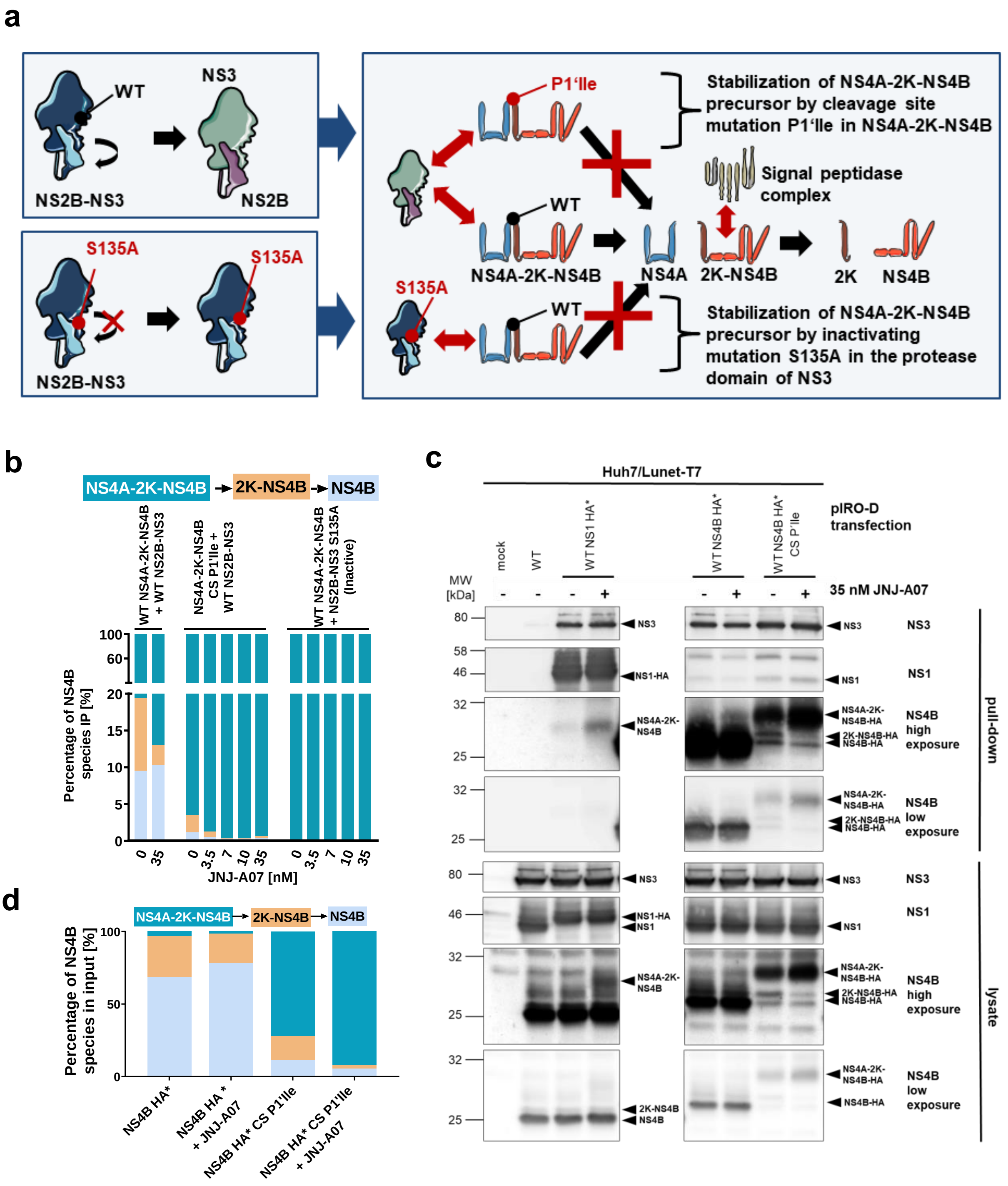

Supplementary Fig. 5 | JNJ-A07 is specifically targeting the interaction between NS4A-2K-NS4B and NS2B/NS3.

a, Schematic representation of the two approaches used to stabilize the NS4A-2K-NS4B precursor. Huh7-T7 cells stably express NS2B-NS3 WT that is cleaved by the NS3 protease domain to form the NS2B/NS3 complex (top left panel). This protease can cleave the transiently expressed NS4A-2K-NS4B WT precursor at the 2K-NS4B cleavage junction giving rise to mature NS4A and to the 2K-NS4B intermediate. The host signal peptidase complex

cleaves off the 2K-peptide from the latter, but is unable to perform this cleavage on the NS4A-2K-NS4B precursor (right panel, middle lane). Two approaches to stabilize the precursor were used. First, the insertion of a cleavage site mutation (P1'Ile) at the 2K-NS4B junction profoundly impairing cleavage at this site (right panel, top lane). Second, expression of a protease dead NS2B-NS3 mutant (S135A) (bottom left panel) unable to cleave the NS4A-2K-NS4B precursor and thus, preventing also signalase mediated cleavage (right panel, lower lane). **b**, Western blot quantification of NS4B-containing species in the pull-down samples (n=3 independent experiments) Representative image shown in Fig. 4d. **c**, Comparison of NS1-HA and NS4B-HA pulldown in the pIRO-D system. 24 h after seeding, Huh7/Lunet T7 cells were transfected with indicated constructs and 4 h later, a medium change was performed and treatment with either JNJ-A07 or solvent control was started. After another 18 h the cells were harvested. HA-immunoprecipitation was performed according to the standard protocol. Western blot (n=1) shows samples before and after HA-precipitation. **d**, Relative quantification of NS4B species in total lysates (refers to c).

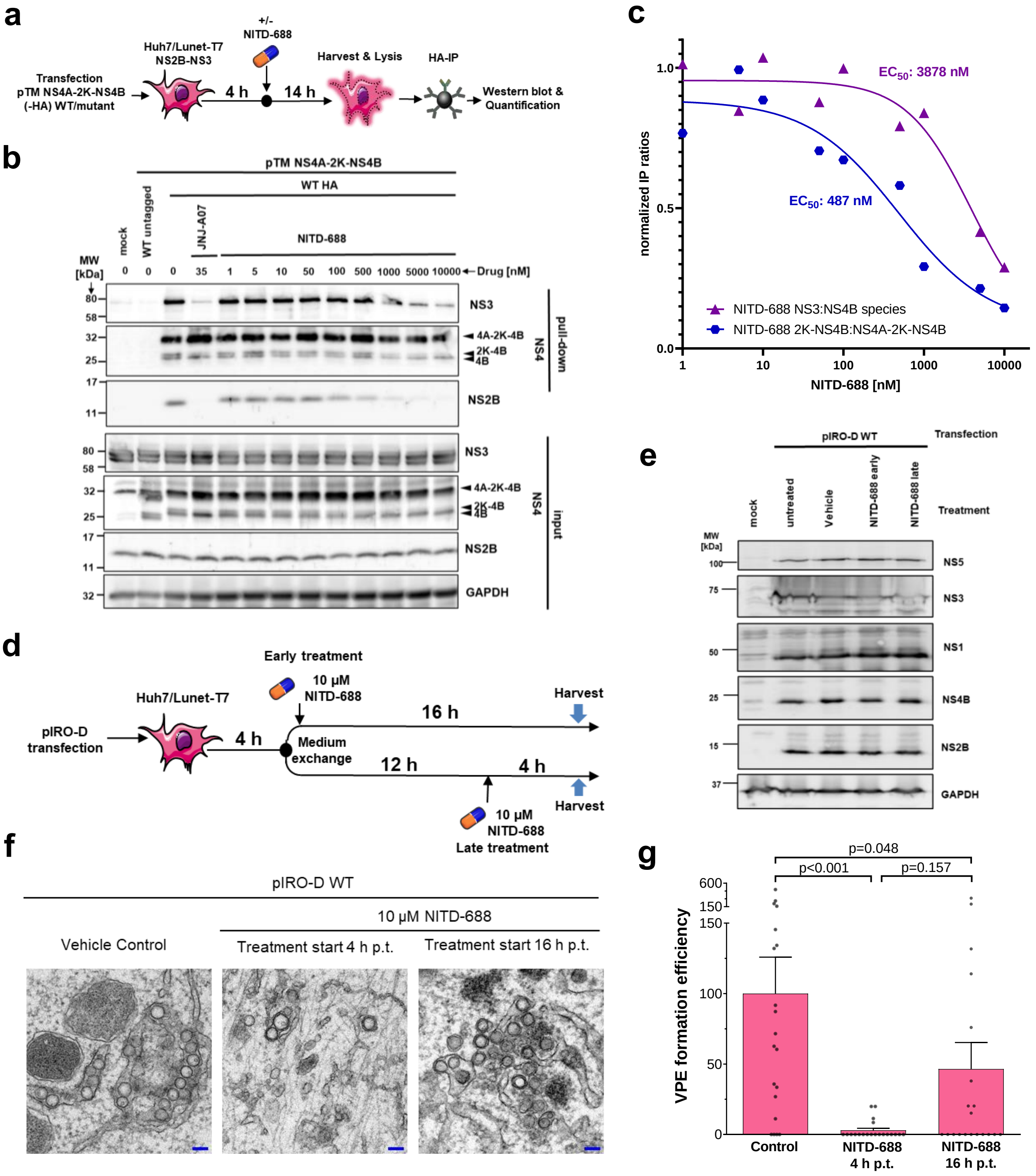

Supplementary Fig. 6 | Similarity of the mechanism-of-action of NITD-688 and JNJ-A07. **a**, Experimental approach: Huh7/Lunet-T7 cells stably expressing DENV NS2B-NS3 were transfected with constructs encoding NS4A-2K-NS4B(-HA). 4 h after transfection, cells were treated with indicated concentrations of NITD-688 (or JNJ-A07 as control) and harvested 14 h later. **b**, Cell lysates were subjected to HA-specific pull-down and analyzed by western blot (n=1). Input and pull-down samples are shown. GAPDH served as loading control. **c**, The western blot shown in (b) was quantified and IP-ratios specified on the bottom were calculated. Dose-response curves were fitted and displayed EC_{50} values were calculated. **d,** Experimental approach to investigate whether NITD-688 influences VPs formation and whether the timing of treatment is critical for this. **e,** No detectable effect of NITD-688 on polyprotein processing. GAPDH served as loading control. **f,** Representative electron micrographs showing VPs. Scale

bar = 100 nm. **g,** Quantitative analysis of electron micrographs. Analogous to the results with JNJ-A07, NITD-688 (10 µM) showed a highly significant reduction of VPEs when treatment was applied early, which was less pronounced when treatment was applied late after transfection. For the plotted data (mean and SEM), 20 cell profiles per sample were analyzed from a single data set. Reported p-values were calculated using Dunn's multiple comparison test, performed after a Kruskal-Wallis test.

Supplementary References

1 Goethals, O. *et al.* Blocking NS3–NS4B interaction inhibits dengue virus in non-human primates. *Nature* **615**, 678-686 (2023).

2 Kaptein, S. J. F. *et al.* A pan-serotype dengue virus inhibitor targeting the NS3–NS4B interaction. *Nature* **598**, 504-509 (2021).
