# Supplementary material for: Pan-serotype dengue virus inhibitor JNJ-A07 targets NS4A-2K-NS4B interaction with NS2B/NS3 and blocks replication organelle formation
Source: Nat Commun. 2024 Jul 19;15:6080. doi: 10.1038/s41467-024-50437-3 (PMC11271582; doi:10.1038/s41467-024-50437-3)
Supplement: Supplementary file 3 — Reporting Summary [file 41467_2024_50437_MOESM3_ESM.pdf]

nature portfolio

Corresponding author(s): R. Bartenschlager

Last updated by author(s): Jun 14, 2024

Reporting Summary

Nature Portfolio wishes to improve the reproducibility of the work that we publish. This form provides structure for consistency and transparency in reporting. For further information on Nature Portfolio policies, see our Editorial Policies and the Editorial Policy Checklist.

Statistics

For all statistical analyses, confirm that the following items are present in the figure legend, table legend, main text, or Methods section.

n/a	Confirmed	
☐	☒	The exact sample size (*n*) for each experimental group/condition, given as a discrete number and unit of measurement
☐	☒	A statement on whether measurements were taken from distinct samples or whether the same sample was measured repeatedly
☐	☒	The statistical test(s) used AND whether they are one- or two-sided<br>*Only common tests should be described solely by name; describe more complex techniques in the Methods section.*
☒	☐	A description of all covariates tested
☒	☐	A description of any assumptions or corrections, such as tests of normality and adjustment for multiple comparisons
☐	☒	A full description of the statistical parameters including central tendency (e.g. means) or other basic estimates (e.g. regression coefficient) AND variation (e.g. standard deviation) or associated estimates of uncertainty (e.g. confidence intervals)
☐	☒	For null hypothesis testing, the test statistic (e.g. *F*, *t*, *r*) with confidence intervals, effect sizes, degrees of freedom and *P* value noted<br>*Give P values as exact values whenever suitable.*
☒	☐	For Bayesian analysis, information on the choice of priors and Markov chain Monte Carlo settings
☒	☐	For hierarchical and complex designs, identification of the appropriate level for tests and full reporting of outcomes
☐	☒	Estimates of effect sizes (e.g. Cohen's *d*, Pearson's *r*), indicating how they were calculated

Our web collection on statistics for biologists contains articles on many of the points above.

Software and code

Policy information about availability of computer code

Data collection	Immunofluorescent images to study the subcellular colocalization between NS3, NS4B and PAL compounds were performed on a Leica SP8 inverted confocal microscope (Leica, Wetzlar, Germany). Fluorescent microscopic imaging for the assessment of transfection efficiency was performed on a Nikon Eclipse Ti microscope using a 20x air objective. Autoradiography data were collected with a BioMax TranScreen LE intensifying screen (Kodak, Rochester, NY, USA) in combination with a Personal Molecular Imager FX (Bio-Rad, Hercules, CA, USA). Western blot data were obtained using a chemoluminescence imager (ECL ChemoCam Imager, Intas Science Imaging Instruments GmbH,Göttingen, Germany). In-gel fluorescence was recorded using a LICOR Odyssey imaging system (LI-COR Biosciences, Lincoln, NE, USA<br>Electron micrographs were collected with a JEM-1400 transmission electron microscope (JEOL, Akishima, Japan).
Data analysis	Western blot data were quantified using the ImageJ2 software package (ImageJ version 2.1.0/1.53j; Wayne Rasband and contributors, National Institutes of 875 Health, USA). Pearson correlation coefficients were determined using the ImageJ plugin Coloc2. Transfection efficiencies were calculated using a custom ImageJ2 macro according to the procedure described in Goellner, S. et al. (Replication-Independent Generation and Morphological Analysis of Flavivirus Replication Organelles. STAR protocols 1, 100173 [2020]). Statistical analyses were performed using GraphPad Prism version 8.4.3. The tests used and corrections for multiple testing are specified in the legends of the respective figures. The same program was also used to create the scientific graphs of this work.

For manuscripts utilizing custom algorithms or software that are central to the research but not yet described in published literature, software must be made available to editors and reviewers. We strongly encourage code deposition in a community repository (e.g. GitHub). See the Nature Portfolio guidelines for submitting code & software for further information.

Data

Policy information about availability of data

All manuscripts must include a data availability statement. This statement should provide the following information, where applicable:

- Accession codes, unique identifiers, or web links for publicly available datasets
- A description of any restrictions on data availability
- For clinical datasets or third party data, please ensure that the statement adheres to our policy

All data supporting the findings of this study are available within the article. Source data are provided with this paper. The source data file contains the raw data on which the graphs and diagrams are based, uncropped versions of any gels and blots shown in the figures as well as the replicates of analyzed gels and blots not shown in the figures. In some figures, basic templates obtained from the Servier Medical Art Library (https://smart.servier.com/) were used.

Research involving human participants, their data, or biological material

Policy information about studies with human participants or human data. See also policy information about sex, gender (identity/presentation), and sexual orientation and race, ethnicity and racism.

Reporting on sex and gender	NA
Reporting on race, ethnicity, or other socially relevant groupings	NA
Population characteristics	NA
Recruitment	NA
Ethics oversight	NA

Note that full information on the approval of the study protocol must also be provided in the manuscript.

Field-specific reporting

Please select the one below that is the best fit for your research. If you are not sure, read the appropriate sections before making your selection.

☒ Life sciences ☐ Behavioural & social sciences ☐ Ecological, evolutionary & environmental sciences

For a reference copy of the document with all sections, see nature.com/documents/nr-reporting-summary-flat.pdf

Life sciences study design

All studies must disclose on these points even when the disclosure is negative.

Sample size	Sample size was not pre-determined. No statistical methods were used to predetermine sample sizes. For Western blot quantifications, the experimental design dictates that only one data point can be collected per sample and experimental execution. For the micrographs, we chose a sample size that, in our opinion, is securely sufficient to represent the variability within the experiment. Sample sizes are stated in the figure legends when applicable.
Data exclusions	No data points were excluded from the datasets in this study.
Replication	Biological replicates were performed on separate days. All attempts at replication were consistent and reflect the intra and inter variability. Three or more independent biological experiments were performed for all experiments presented in main figures except for the determination of vesicle packets formation efficiency, for which two independent biological experiments were performed. For the data in the supporting figures, different sample sizes (ranging from n=1 to n=3) were used, which are indicated in the respective figure legends.
Randomization	Since the work presented focuses exclusively on in vitro experiments, randomization was not relevant, as no allocation to treatment groups was required.
Blinding	For the in vitro experiments used in this study, blinding was not applicable as no experimental treatment groups were used where the quality of the outcome could be influenced.

Reporting for specific materials, systems and methods

We require information from authors about some types of materials, experimental systems and methods used in many studies. Here, indicate whether each material, system or method listed is relevant to your study. If you are not sure if a list item applies to your research, read the appropriate section before selecting a response.

Materials & experimental systems

n/a	Involved in the study
☐	☒ Antibodies
☐	☒ Eukaryotic cell lines
☒	☐ Palaeontology and archaeology
☒	☐ Animals and other organisms
☒	☐ Clinical data
☒	☐ Dual use research of concern
☒	☐ Plants

Methods

n/a	Involved in the study
☒	☐ ChIP-seq
☒	☐ Flow cytometry
☒	☐ MRI-based neuroimaging

Antibodies

Antibodies used

anti DENV NS2B rabbit polyclonal antibody (GTX124246, Lot #40751, GeneTex)
anti DENV NS3 mouse monoclonal antibody (GTX629477, Clone GT2811, Lot# 42961, GeneTex)
anti DENV NS4B rabbit polyclonal antibody (GTX124250, Lot# 40779, GeneTex)
anti ZIKV NS4B rabbit polyclonal antibody (GTX133321, Lot# 42697, GeneTex)
anti-GAPDH, G-9 (sc-365062, Lot # I2320, Santa Cruz Biotechnology)
anti β-Actin (A5541, clone AC-15, Lot # 0000090942, Sigma-Aldrich)
anti-HA.11 epitope tag, clone: 16B12 (lot #: B276381, BioLegend)

Mouse monoclonal anti-HA agarose beads, clone HA-7 (A2095, Lot# 119M4756V, Sigma-Aldrich)

anti DENV NS1 rabbit polyclonal antibody (homemade)
anti DENV NS3 rabbit polyclonal antibody (homemade)
anti DENV NS4B rabbit polyclonal antibody (homemade)
anti DENV NS5 rabbit polyclonal antibody (homemade)

Validation

Antibodies were validated in previous studies (Miller et al., 2006; Chatel-Chaix et al., 2015) or validated by the manufacturers.

Eukaryotic cell lines

Policy information about cell lines and Sex and Gender in Research

Cell line source(s)

Human hepatocellular carcinoma cells (Huh-7; Nakabayashi et al., Cancer Research 1982) were obtained from Prof. Heinz Schaller (Center for Molecular Biology Heidelberg (ZMBH), Germany) and served as the founders for the other cell lines listed below.

Huh7/Lunet cells (Huh7 cell-derived clone that was cured of stable HCV replicon expression by selective substance treatment) were reported in Friebe et al., JVI, 2005.

T7-expressing cell lines Huh7-T7 and Huh7/Lunet-T7 were reported in Appel et al., JVI, 2005.

Huh7-T7 DENV2 NS2B-NS3 and Huh7/Lunet-T7 DENV2 NS2B-NS3 were reported in Chatel-Chaix et al., JVI 2015.

Huh7/Lunet-T7 DENV-2 NS1-HA cells were reported in Cortese et al., JVI 2021.

Huh7/Lunet-T7 Calnexin-HA cells were generated from Huh7/Lunet-T7 by lentiviral transduction as reported in Chatel-Chaix et al., JVI 2015.

Authentication

Cell lines were not regularly authenticated beyond microscopic inspection for cell morphology.

Mycoplasma contamination

Cell lines were regularly tested for mycoplasma contamination.

Commonly misidentified lines
(See ICLAC register)

None of the commonly misidentified cell lines were used.
